# Prevalence of Anti-Neu5Gc Antibodies in Patients with Hypothyroidism

**DOI:** 10.1155/2014/963230

**Published:** 2014-06-09

**Authors:** Phaedra Eleftheriou, Stavros Kynigopoulos, Alexandra Giovou, Alexandra Mazmanidi, John Yovos, Petros Skepastianos, Eleni Vagdatli, Christos Petrou, Dafni Papara, Maria Efterpiou

**Affiliations:** ^1^Department of Medical Laboratory Studies, School of Health and Medical Care, Alexander Technological Educational Institute of Thessaloniki, TEI Campus Sindos, 57400 Thessaloniki, Greece; ^2^1st Internal Medicine Department, AHEPA University General Hospital of Thessaloniki, St. Kyriakidi 1, 54636 Thessaloniki, Greece; ^3^Aristotle University of Thessaloniki, St. Kyriakidi 1, 54636 Thessaloniki, Greece

## Abstract

*Background*. N-Glycolylneuraminic acid (Neu5Gc) is a sialic acid synthesized by animals, but not by humans or birds. However, it can be incorporated in human cells and can trigger immune response. In the present study, we detected anti-Neu5Gc antibodies in samples of the general population and of patients suffering from hypothyroidism/Hashimoto's disease, which is known to have autoimmune origin. *Methods*. Antibodies were measured using enzyme-immunosorbent techniques. *Results*. Serum anti-Neu5Gc IgG antibodies were higher in patients with hypothyroidism (mean: 14.8 ± 15.9 **μ**g/mL, median: 10.0 **μ**g/mL, *P* = 0.0003, Mann-Whitney) and even higher in the group with Hashimoto's thyroiditis (mean: 31.1 ± 16.3 **μ**g/mL, median: 27.2 **μ**g/mL, P = 0.0000, Mann-Whitney) compared to the general population (mean: 5.3 ± 4.7  **μ**g/mL, median : 4 **μ**g/mL). All anti-TPO positive samples had anti-Neu5Gc antibody concentration higher than the mean value of the general population while anti-TPO concentration was increased as anti-Neu5Gc concentration increased. Low concentrations of IgA and IgM antibodies were measured in both general population and patient groups. *Conclusion*. The increased values of anti-Neu5Gc antibodies in patients with hypothyroidism/Hashimoto's disease and the correlation of anti-TPO incidence with increased anti-Neu5Gc concentration raise the possibility of an association between anti-Neu5Gc antibody development and autoimmune hypothyroidism.

## 1. Introduction

N-Glycolylneuraminic acid (Neu5Gc) is a sialic acid which is synthesized by animals, but not by humans or birds because of the lack of the enzyme CMP-Neu5Ac hydroxylase which converts Neu5Ac to Neu5Gc [1]. However, it can be incorporated in human cells after entering the body [2–11]. According to Bardor et al. [11], incorporation of Neu5Gc involves a pinocytic/endocytic procedure and the action of lysosomal sialidase as well as lysosomal sialic acid transporter. Such incorporation was detected in human cells cultivated in vitro in animal derived media [2, 11]. However, Neu5Gc accumulation to human cells may arise from dietary sources such as red meat and milk products [2]. Interestingly, Neu5Gc containing molecules trigger immune system response. Antibodies recognizing Neu5Gc have been detected in individuals of the general population even if they have not received animal products intravenously [1, 12, 13]. Although low concentrations of anti-Neu5Gc antibodies do not seem to have any effect on Neu5Gc containing cells, it was shown that human sera containing naturally occurring high levels of anti-Neu5Gc IgG antibodies could trigger cell cytotoxicity [12]. This observation raises the concept that Neu5Gc may be responsible for the development of human diseases associated with chronic inflammation or autoimmune diseases. Although this assumption has been previously stated by other investigators [1], no such evidence has been gathered till now concerning autoimmune diseases. According to our knowledge, this is the first study investigating the association of anti-Neu5Gc antibodies with autoimmune diseases such as hypothyroidism.

The incidence of clinical hypothyroidism is 0.5–1.9% in women and <1% in men and that of subclinical hypothyroidism varies between 3–13.6% in women and 0.7–5.7% in men [14]. About 50% of these cases are of autoimmune origin [14]. Although the deeper causes of the disease have not been clarified yet, there is evidence that a genetic propensity, combined with environmental factors, promotes the disease. A hereditary has been observed by studying families as well as twins. However, the finding that the concordance rate is no more than 50% even with identical twins strongly indicates other important factors [15–20]. Hashimoto's disease is the most common cause of primary hypothyroidism. Autoantibodies against thyroid peroxidase and/or thyroglobulin are detected in the serum and can cause gradual destruction of follicles in the thyroid gland.

Interestingly, both autoantigens related to Hashimoto disease are glycoproteins and N-linked carbohydrates containing sialic acids have been detected in both molecules [21, 22]. Alteration in glycosylation may alter antigenic activity as well as interaction with other molecules. There are several investigations examining the involvement of the carbohydrate part in autoimmunity. Although the necessity of the carbohydrate group for the interaction with the autoantibodies is not supported by these researches, the reported observations presented in the studies showed that glycopeptides prepared from human thyroglobulin inhibited the immunoreaction between native thyroglobulin and autoantisera [23]. However, desalination of thyroglobulin either increased or decreased reactivity with autoantibodies of patients' sera, in a manner depending on the antisera and thyroglobulin source [23]. A slight but not statistically significant decrease in auto-antibody-binding capacity following desalination of human recombinant TPO enzyme has also been mentioned [22]. It is well known that once a conjugate between a nonantigenic and a foreign antigenic molecule triggers immune response, antibodies recognizing the antigenic as well as “non-antigenic” parts are formed [24, 25]. Under this concept, incorporation of Neu5Gc could be responsible for the initial formation of autoantibodies in autoimmune thyroid disease.

In the present study, as a first step in the examination of a probable relationship between the occurrence of anti-Neu5Gc antibodies and the development of autoimmune thyroiditis, we investigated the prevalence of anti-Neu5Gc antibodies in the serum of people suffering from hypothyroidism/Hashimoto thyroiditis and compared it to that of general population and people suffering from rheumatoid arthritis.

## 2. Materials and Methods

### 2.1. Materials

#### 2.1.1. Biological Samples

Blood serum from 50 individuals (24 men and 26 women) of the general population, 48 serum samples from patients (3 men and 45 women) suffering from hypothyroidism, 35 samples from patients suffering from Hashimoto thyroiditis (women), and 25 samples from patients suffering from rheumatoid arthritis (1 man, 24 women) were used. The age of all groups varied between 35 and 65 years old. Blood samples were allowed to clot for thirty minutes in room temperature and centrifuged for 15 minutes at 2500 ×g. Serum aliquots were separated and kept at −20°C until use. All donors were informed about the research and gave samples voluntarily.

#### 2.1.2. Chemical and Biochemical Reagents

All chemical reagents were of analytical grade and were products of Merck, Fluka, and Riedel de Haen. Microtiter 96 well, flat bottom, high binding plates were purchased by Greiner Bio-One. Glycolylneuraminic acid (Neu5Gc) was a product of Sigma. Peroxidase-conjugated, goat, and anti-human IgG or IgM or IgA were purchased by AbD Serotec.

5235P seasonal human H1N1 influenza virus hemagglutinin peptide and rabbit anti-5235P antibody were purchased by ΨProSci and peroxidase-conjugated anti-rabbit IgG was purchased by Sigma. TG-Ab ELISA test and TPO-Ab ELISA test were products of IBL International.

### 2.2. Methods

#### 2.2.1. Measurement of Anti-Neu5Gc Antibodies

For the determination of anti-Neu5Gc antibodies, 96 well, flat bottom, high binding microtiter plates were used. The wells were coated with Neu5Gc antigen by adding 100 *μ*L of a solution containing 1.6 *μ*g/mL Neu5Gc in 0.05 M carbonate buffer pH 9.6. Following 16 h incubation at room temperature, the solution was washed out. At the second stage, 200 *μ*L of blocking solution containing 0.1% egg albumin in PBS (137 mM NaCl, 2.7 mM KCl, 10 mM Na_2_HPO_4_, 2 mM KH_2_PO_4_) pH 7.2 was added to each well and was incubated for 2 h. At the third stage, 100 *μ*L of appropriate serum dilution in PBST (PBS, 0.05% Tween-80) was added to each well and was incubated for 1 h at room temperature. Three different dilutions 1 : 100, 1 : 500, and 1 : 1000 were first used. Dilution 1 : 500 was selected as the most appropriate for final application of the method. The 1/500 serum dilution was selected because it enabled a clear distinction between the concentration of the samples (absorptions between 0.400 and 1.800, within the optimal absorption scale of microtiter plate reader, and sample absorption nearly within the linear part calibration curve). After aspiration of the supernatant and three washes with PBST and double distilled water, 100 *μ*L of peroxidase-conjugated anti-human IgG (1 : 25000) was added to each well. Following 1 h incubation, the supernatant was rejected and after washing with PBST and double-distilled water, peroxidase substrate ABTS was added to the wells. The colored product produced was measured at an ELISA reader at 405 nm. All samples were measured in duplicate. In all cases the difference between two measurements of the same sample was less than 10%. In a similar way, HRP conjugated goat anti-human IgM or IgA (AbD serotec) was used as secondary antibody for the measurement of anti-Neu5Gc IgM and IgA antibodies.

In each measurement, ten wells of the plate were coated with the 5235P oligopeptide antigen by the addition of 100 *μ*L of a solution containing 1.6 *μ*g/mL of 5235P antigen in 0.05 M carbonate buffer pH 9.6 in each well. At the third stage of the procedure, five different dilutions of rabbit anti-5235P antibody in PBST were used. The five dilutions (5, 10, 100, 200, and 500 ng/mL) were added in duplicate. Peroxidase conjugated anti-rabbit IgG was added at the fourth stage of the procedure. Absorbance measurement of the wells was used as a verification of the successful application of the whole procedure. Moreover, absorbance measurements (A405) of the wells with known anti-5235P antibody concentration were used for the preparation of a calibration curve. This calibration curve can be used for the measurement of antibody concentration in a way similar to that of standard dilution curve of purified human IgG which has been used by other investigators. Since the aim of the study was not the measurement of the absolute anti-Neu5Gc concentration but the comparison between the amount of anti-Neu5Gc present in general population and in patients suffering from hypothyroidism, a relative concentration could be used as an acceptable tool. Neu5Ac-coated microtiter plates were used for evaluating the cross-reaction with Neu5Ac.

#### 2.2.2. Measurement of Anti-TG Antibodies [26]

Anti-TG antibodies were measured using the Enzyme Immunoassay kit purchased by IBL International (RE75501). The method is a solid phase enzyme-immunosorbent assay (ELISA) based on the sandwich principle. According to the method, expected values for the normal population are as follows: mean: 35 IU/mL (SD = 37.5), mean + 2  SD: 110 IU/mL, median: 25 IU/mL, and 95% of samples upper limit: 60 IU/mL. Concentration values lower than 85 IU/mL are considered negative, 85–125 IU/mL are considered equivocal, and higher than 125 IU/mL are considered positive.

#### 2.2.3. Measurement of Anti-TPO Antibodies [26]

Anti-TPO antibodies were measured using the Enzyme Immunoassay kit purchased by IBL International (RE75511). The method is a solid phase enzyme-immunosorbent assay (ELISA) based on the sandwich principle. According to the method, expected values for the normal population are as follows: mean: 55 IU/mL (SD = 82.5), mean + 2 SD: 220 IU/mL, median: 35 IU/mL, and 95% of samples upper limit: 110 IU/mL. Concentration values lower than 85 IU/mL are considered negative, 85–125 IU/mL are considered equivocal, and higher than 125 IU/mL are considered positive.

#### 2.2.4. Statistical Analysis

Statistical analysis of data was carried out using PAST programme [27, 28]. Shapiro-Wilk *W* test [29, 30] was used to check normality of distribution of values of anti-Neu5Gc concentration in general population or in patients suffering from hypothyroidism. The Mann-Whitney nonparametric test for independent samples was used to evaluate the significance of difference of concentrations of anti-Neu5Gc antibodies in the two groups [29, 31]. Chi square test and Fisher's exact test [32] were used to evaluate statistical significance of the difference in percentage of positives between the two groups.

## 3. Results 

### 3.1. Prevalence of Anti-Neu5Gc Antibodies in General Population

Anti-Neu5Gc IgG antibodies were determined in almost all samples of the general population with an average concentration of 5.3 *μ*g/mL (SD = 4.1 *μ*g/mL). The concentrations measured varied between 0 and 24 *μ*g/mL (Table 1). The values do not follow a normal distribution according to Shapiro-Wilk *W* test (*W* = 0.870, *P* = 0.0001) with a median value of 4 *μ*g/mL. Half the samples of general population (50%) had anti-Neu5Gc concentration <4 *μ*g/mL, 81% of the population had anti-Neu5Gc concentration ≤9.4 *μ*g/mL (≤ mean + 1 SD), and 94% had concentration ≤13.5 *μ*g/mL (≤ mean + 2 SD), while 98% had concentration <16 *μ*g/mL (Figure 2).

**Figure 1:**
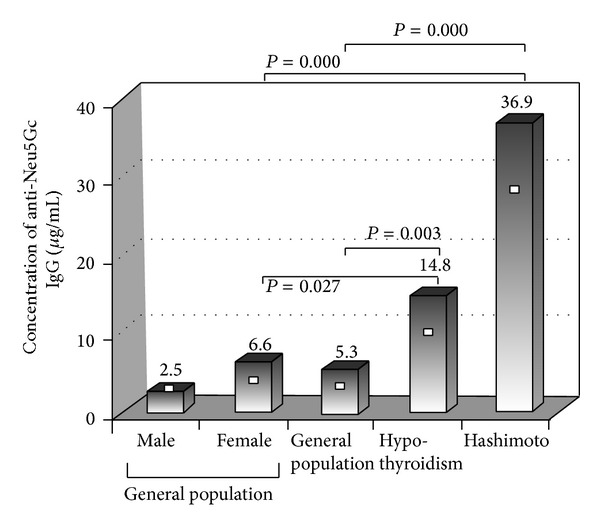
Concentration of anti-Neu5Gc antibodies in general population and in patients groups suffering from hypothyroidism and more specifically by Hashimoto disease. Mean values are presented with bars. White squares (□) represent the median value. *P* values were driven by application of Mann-Whitney test.

**Figure 2:**
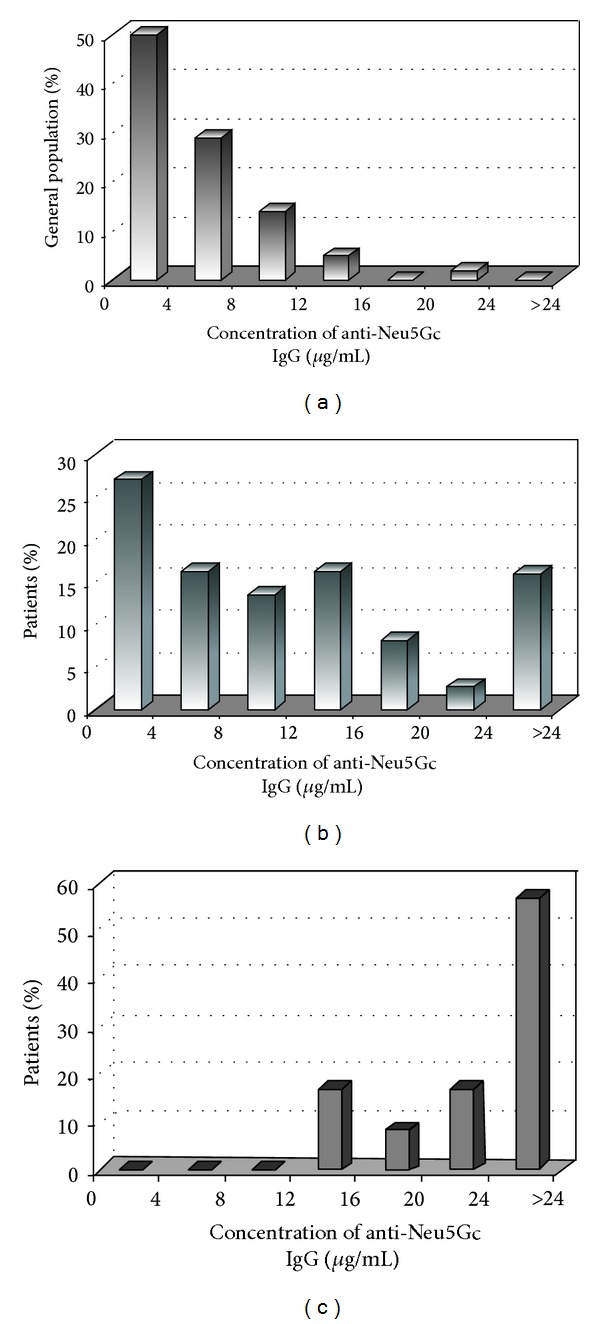
Distribution of anti-Neu5Gc IgG antibodies in general population (a) and in people suffering from hypothyroidism (b) or more specifically by Hashimoto disease (c).

A tendency for higher anti-Neu5Gc concentration was found in female (average 6.6 *μ*g/mL, SD = 6.7 *μ*g/mL, median = 4.0 *μ*g/mL, max. = 24 *μ*g/mL) compared to the male individuals (average 2.5 *μ*g/mL, SD = 2.9 *μ*g/mL, median = 3.0 *μ*g/mL, max. = 10 *μ*g/mL) of the general population (Table 1, Figure 1). However, the difference cannot be considered statistically significant (*P* = 0.336, according to Mann-Whitney *W* test). Interestingly, anti-Neu5Gc concentration in males follows a normal distribution (Shapiro-Wilk test *W* = 0.9465, *P* = 0.4035), while anti-Neu5Gc values in females do not (Shapiro-Wilk test *W* = 0.8696, *P* = 0.01751).

Low concentrations of anti-Neu5Gc IgM, 2.5 ± 27 *μ*g/mL, and IgA antibodies, 2.3 ± 0.8 *μ*g/mL were also detected in the general population (Tables 3 and 4).

### 3.2. Prevalence of Anti-Neu5Gc, Anti-TG, and Anti-TPO Antibodies in the Group of People Suffering from Hypothyroidism

Serum anti-Neu5Gc antibodies were higher in patients with hypothyroidism as compared to the general population (mean: 14.8 ± 15.9 *μ*g/mL, median: 10.0 *μ*g/mL versus mean: 5.3 ± 4.7 *μ*g/mL, median: 4 *μ*g/mL, *P* = 0.0003, Mann-Whitney) as well as compared to the female group of the general population (mean: 6.6 ± 6.7 *μ*g/mL, median: 4.0 *μ*g/mL, *P* = 0.0273 Mann-Whitney) (Table 1).

If samples with antibody concentration greater than 13.5 *μ*g/mL (> mean + 2 SD of general population) are considered positive, the prevalence of positive anti-Neu5Gc samples in the general population was 6.0% and in the hypothyroidism population was 42.1%. The observed difference was found to be statistically significant (odd ratio = 0.089, 95% CI = 0.024−0.381, *P* < 0.000 according to Fisher's exact test, Table 2). When the samples of the group of patients were tested for interaction with the naturally occurring sialic acid Neu5Ac, a degree of interaction was observed in all anti-Neu5Gc positive samples. However, the concentration of antibodies recognizing Neu5Gc was always higher than that interacting with Neu5Ac although a linear relation between the two concentrations was not observed. A ratio between the concentrations of antibodies recognizing Neu5Gc to the concentration of that interacting with Neu5Ac in a positive sample could be 14.1 *μ*g/mL versus 4 *μ*g/mL. This probably indicates the presence of cross-reaction or nonspecific interaction with Neu5Ac but does not diminish the significance of increased concentrations of Neu5Gc-recognizing antibodies in patients with hypothyroidism versus general population.

**Figure 3:**
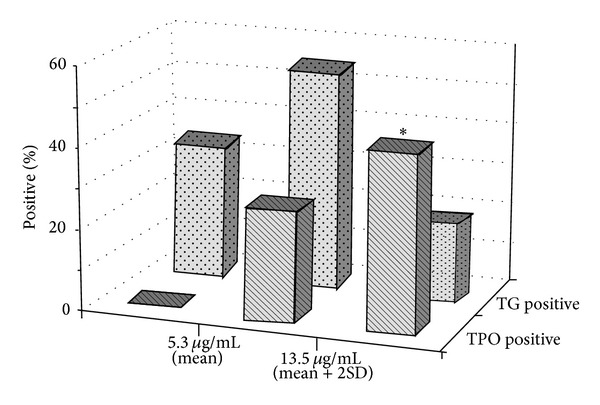
Prevalence of samples positive to anti-TG and anti-TPO antibodies in patients with different anti-Neu5Gc antibody concentrations. *odd ratio = 0, Fisher's exact *P* = 0.0002 when compared with the group with anti-Neu5Gc < 5.3 *μ*g/mL. 5.3 *μ*g/mL = mean anti-Neu5Gc concentration in general population, and 13.5 *μ*g/mL = mean + 2 SD anti-Neu5Gc concentration of general population.

A proportion of 31.3% and 27% of the patients with hypothyroidism were found positive to anti-TG or anti-TPO, respectively, while 47.9% of the patients were positive in one or both thyroid specific autoantibodies. All anti-TPO positive samples had anti-Neu5Gc antibody concentration higher than the median and the mean value of general population. The percentage of anti-TPO positive samples increased as anti-Neu5Gc concentration was increased (Figure 3). The presence of anti-TG antibodies was not correlated to the concentration of anti-Neu5Gc. Anti-TG antibodies were also detected in samples with anti-Neu5Gc concentration lower than the mean value of the general population, although a greater prevalence of anti-TG positive samples was observed in the group of patients with anti-Neu5Gc concentration varying between mean and mean + 2 SD.

Recent triggering of immunologic response and a probable involvement of mucosal tissue in immune reaction was evaluated by the measurement of IgM and IgA antibodies, respectively. The determination revealed a statistically significant increase of 32% (Mann-Whitney test, *P* = 0.00285) in IgM concentration and a 26.1% not statistically significant increase in IgA concentration compared to general population (Tables 3 and 4).

### 3.3. Prevalence of Anti-Neu5Gc, Anti-TG, and Anti-TPO Antibodies in People Suffering from Hashimoto Disease

Nearly all patients suffering from Hashimoto thyroiditis were positive to anti-Neu5Gc antibodies (97.1%, OR: 0.002, *P* < 0.000) having anti-Neu5Gc concentration higher than 13.5 *μ*g/mL (mean + 2 SD of the general population). 57.1% of the patients had anti-Neu5Gc concentration higher than 24 *μ*g/mL, which was the maximum value observed in general population. All the patients were positive to anti-TG or anti-TPO antibodies. 80% of the patients were anti-TPO positive while 51.4% were anti-TG positive.

**Figure 4:**
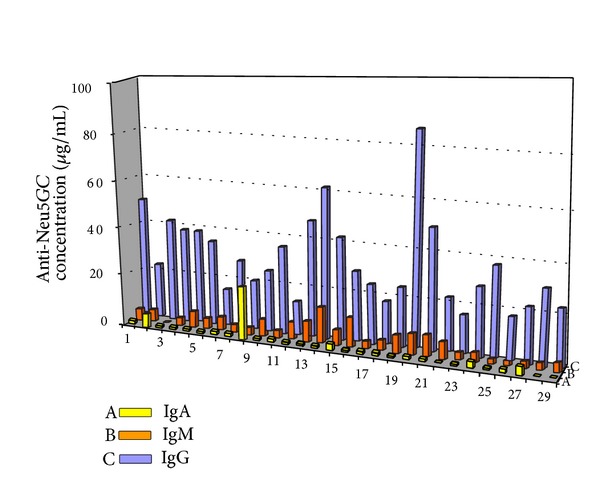
Concentration of anti-Neu5Gc IgA (A: yellow), IgM (B: orange), and IgG (C: blue) antibodies in patients suffering from Hashimoto thyroiditis.

Determination of anti-Neu5Gc IgM and IgA antibodies revealed a statistically significant 50% increase (Mann-Whitney test, *P* = 0.00080) in IgM concentration and a 13.0% decrease (Mann-Whitney test, *P* = 0.00285) in IgA concentration compared to the general population (Tables 3 and 4, Figure 4).

### 3.4. Prevalence of Anti-Neu5Gc Antibodies in People Suffering from Rheumatoid Arthritis

Preliminary results concerning the prevalence of anti-Neu5Gc antibodies in people suffering from Rheumatoid arthritis (25 individuals, 1 men, 24 women) did not show statistically significant difference compared to the general population (average = 4.5, SD = 5.2, median = 4.0, *P* = 0.3647, Mann-Whitney test). Very low anti-Neu5Gc concentration was detected even in samples with considerably high RA factor values, even higher than 200 IU/mL, or/and CRP (C-reactive protein) values higher than 20 mg/L.

## 4. Discussion

The average anti-Neu5Gc concentration identified in the general population (5.3 ± 4.2 *μ*g/mL, median: 4 *μ*g/mL, max. 24 *μ*g/mL) was in accordance with that mentioned by other scientists. In general, mean values varying from 0.3 to 6.0 *μ*g/mL [13] and sample concentrations varying from minimum of 0.09 to a maximum of 75 *μ*g/mL have been detected in apparently healthy individuals [2, 12, 13]. Variations in concentration are observed depending on the type of Neu5Gc conjugate bound at the bottom of ELISA plate [13]. Polyacrylamide conjugated Neu5Gc [2, 12] and various Neu5Gc-containing carbohydrate molecules [13] have been previously used. In the present study, Neu5Gc molecule was directly bound to Microlon resin of the microtiter plates. There is no literature concerning anti-Neu5Gc concentration in male and female population. However, since hypothyroidism is mainly a women's disease, the prevalence of anti-Neu5Gc antibodies in the female group of general population was substantial for the evaluation of the results concerning patients suffering from hypothyroidism and Hashimoto thyroiditis. Although the observed difference of anti-Neu5Gc concentration between male and female was not statistically significant, a tendency for higher anti-Neu5Gc concentrations was found in female (average 6.6 *μ*g/mL, SD = 6.7 *μ*g/mL, median = 4.0 *μ*g/mL, max. = 24 *μ*g/mL) compared to the male individuals (average 2.5 *μ*g/mL, SD = 2.9 *μ*g/mL, median = 3.0 *μ*g/mL, max. = 10 *μ*g/mL) in the general population. In addition to clinical hypothyroidism with an incidence of 0.5–1.9%, subclinical hypothyroidism is also observed in 3–13.6% of women [14]. Consequently, the greater concentration of anti-Neu5Gc antibodies and the lack of normal distribution in the female group of general population compared to the male group may be related to the greater incidence of individuals with hypothyroidism, probably subclinical one, in the female subgroup.

The hypothyroid patients showed a statistically significant increase in anti-Neu5Gc concentration (14.8 ± 15.9 *μ*g/mL, median: 10.0 *μ*g/mL) which corresponds to more than 2.5-fold higher average value compared to the general population. Since hypothyroidism is more common in women with a ratio women/men varying from 6/1 to 10/1 depending on district and age [33–36], the hypothyroidism group mostly consisted of women (3 men, 45 women). So, the anti-Neu5Gc concentration of the patients was also compared with the values of the female group of the general population. The comparison revealed a statistically significant difference, corresponding to 2.25-fold increase in the hypothyroidism group. Concerning the samples with antibody concentration greater than mean value + 2 SD of general population as positive, the prevalence of positive anti-Neu5Gc samples in the hypothyroidism group was 7-fold greater than that of the general population (42.1% versus 6%, odd ratio = 0.089, *P* < 0.000, Fisher's exact test).

According to the literature, about 50% of hypothyroidism incidences are of autoimmune origin [14]. The role of autoimmunity is supported by the histological findings and by the identification of specific antibodies against thyroid autoantigens [37]. Increased levels of anti-TPO antibodies are usually found in about 95% and anti-TG antibodies in about 60% of the cases of autoimmune hypothyroidism, according to bibliography [14]. In our study, a proportion of 31.3% and 27% of hypothyroid patients were found positive to anti-TG or anti-TPO, respectively, while 47.9% of the patients were positive to one or both thyroid specific autoantibodies indicating autoimmune origin. All anti-TPO positive samples had anti-Neu5Gc antibody concentration higher than the median value of general population, while anti-TPO concentration was increased as anti-Neu5Gc concentration increased.

The increased values of anti-Neu5Gc antibodies in patients with hypothyroidism and the correlation of anti-TPO incidence with increased anti-Neu5Gc concentration raised the concept of a probable association between anti-Neu5Gc antibody development and autoimmune hypothyroidism. The concept was further exploited by the study of a group of patients with Hashimoto's thyroiditis. The obtained results further support the hypothesis of association. All the patients were positive to anti-TG or anti-TPO antibodies with 80% being anti-TPO positive and 51.4% anti-TG positive while nearly all patients (with one exception, 97.1%) were anti-Neu5Gc positive with significantly increased antibody concentrations.

The absence of increased anti-Neu5Gc concentration in individuals with rheumatoid arthritis, which is also considered autoimmune disease [38], indicates that Neu5Gc is not involved in the development of this disease and increased anti-Neu5Gc antibodies are not a common characteristic of all autoimmune diseases. In rheumatoid arthritis, autoantibodies like RA factor which is recognizing the Fc fraction of IgG antibodies and ACPA (anticitrullinated protein antibodies) are detected in about 67% of the patients [39, 40]. Moreover, antibodies against filaggrin, calpastatin, glucose-6-phosphate isomerase, and enolase are also found [41].

Further investigation is needed to elucidate if incorporation of Neu5Gc in TG and TPO molecules is involved in the initiation of autoantibodies development. However, both molecules are glycoproteins with multiple sites of N-glycosylation. Sialic acids are commonly the end monosaccharides of carbohydrate moieties linked to proteins via N-glycosylation of Asn or Arg aminoacid side chains. The extracellular antigenic part of TPO, which is known to be connected to the complement in autoimmune hypothyroidism, contains four sites for Asn N-glycosylation (aminoacid sequence: NXS/T, X different than proline). It has been shown that purified human and human recombinant molecules of TPO contain complex carbohydrates [42] with sialic acids among them [43]. Moreover, it is well known that sialic acids are within carbohydrates of human thyroglobulin [44]. Carbohydrate composition as well as sialic acid concentration differs in thyroglobulin of normal and pathologic thyroid tissue [44]. Neu5Ac is the end sialic acid of 65% of carbohydrates of normal human thyroglobulin. Differences in carbohydrate composition of TG or TPO molecules referred to in the literature may be the result of different contribution of immature molecules in the preparation used for the examination. Although sialic acids in the carbohydrate moieties of TG and TPO molecules could be substituted by Neu5Gc under certain conditions, it needs to be proved for the patients suffering from hypothyroidism. Neu5Gc incorporation in human molecules remains a complex and unknown process. Even when released in the circulation, Neu5Gc is not equally incorporated by all cells [12]. Moreover, even after being inserted in a cell by pinocytosis, it is not expected to substitute all Neu5Ac end-monosaccharides. Carbohydrate analysis of thyroglobulin of porcine origin, where Neu5Gc is naturally synthesised, revealed the presence of nine Neu5Ac and one Neu5Gc end sialic acid in the seven N-linked carbohydrate moieties of the molecule [45]. However, it is not clear how this distribution is controlled. It is obvious that the capability of sialylation is a prerequisite but cannot explain alone a probable Neu5Gc incorporation. Hundreds of sialylated proteins exist in human cells, though not known to be related with autoimmune diseases in most cases. Certain target proteins of autoantibodies in rheumatoid arthritis are also glycoproteins bearing sialic acids.

It may be worth mentioning, however, that, in case of Hashimoto thyroiditis, among the genes associated with increased incidence of the disease, there are two variants involving Arg residue which could be potentially N-glycosylated: TG variant (W1999R) and HLA-DR variant 74R [46, 47].

The study of carbohydrate content of molecules related to Hashimoto thyroiditis in tissues of people suffering from this disease could enlighten the riddle, though it is not so easy to collect samples from the patients since biopsies or tissue extraction is not so common in this kind of patients. Monitoring anti-Neu5Gc concentration during time in the serum of healthy individuals who belong in the high risk group because of a family history or genetic characteristics in correlation to the consumption of red meat and related products would give valuable information concerning the involvement of Neu5Gc in the development of the disease. In an analogue way, the monitoring of anti-Neu5Gc and autoantibody concentration in the group of subclinical hypothyroidism and their progress to disease in relation to practices favoring Neu5Gc incorporation could also be valuable.

The increased concentrations of IgM antibodies in patients with hypothyroidism and the even higher concentrations in patients with Hashimoto's thyroiditis compared to general population indicate recent triggering of immunologic response. In most cases, samples with high anti-Neu5Gc IgG concentration also exhibited relatively high IgM concentration (Figure 4). Since Neu5Gc may be present in foods, food additives, and medicines produced using animal cell lines or culture media [48], repeated triggering is possible. Low anti-Neu5Gc IgA concentrations were detected both in general population and patient's groups. Interestingly, the concentrations detected in patients with Hashimoto's thyroiditis were lower than those of the general population. Production of IgA antibodies belongs in the first line of defense and is commonly observed in immune system response to food derived antigens because of the IgA producing cells of intestinal mucosa [49]. However, low IgA accompanied by high IgM antibodies against food derived antigens has been identified in some groups of patients with food derived diseases such as celiac disease [50]. This may indicate impairment at the first line of defense leading to increase in immunoglobulins of the second line (IgM and IgG).

## 5. Conclusions

The high prevalence of anti-Neu5Gc antibodies in patients with hypothyroidism (42.1%, *P* < 0.000) and in patients with Hashimoto disease (97.1%, *P* < 0.000) compared to the general population (6.0%) and the increased incidence of anti-TPO antibodies in patients with high anti-Neu5Gc concentration raise the concept of a probable association between anti-Neu5Gc antibody development and autoimmune hypothyroidism. Incorporation of the foreign, food derived antigen Neu5Gc in human glycoproteins, such as TPO, could be among the factors involved in the initiation and development of the disease. However, further experiments are needed to elucidate if the development of anti-Neu5Gc antibodies is among the causative factors or a parallel result of certain processes related to the disease. In any case, the development of autoimmune thyroiditis is a multifactorial process probably involving a number of environmental factors combined with genetic susceptibility, as the study of identical twins has already proved.

The prevalence (97.1%) of increased anti-Neu5Gc antibodies in Hashimoto's disease equals and even exceeds the prevalence of anti-TPO antibodies (80% in the present study, 90% according to the literature) which is the most characteristic autoantibody associated with the disease. No matter if increased anti-Neu5Gc antibodies represent a causative factor or a secondary result of the process, increased anti-Neu5Gc concentrations could be associated with increased probability for the development of the disease, especially in people with a family history. Although no association of increased anti-Neu5Gc concentration with rheumatoid arthritis was observed, a probable association with other autoimmune diseases may exist and is currently investigated by our team.

## Figures and Tables

**Table 1:** Concentration of anti-Neu5Gc IgG antibodies.

	*n*	Mean value (*μ*g/mL)	SD	Median value (*μ*g/mL)	Minimum (*μ*g/mL)	Maximum (*μ*g/mL)	*P*
General population	50	5.3	4.2	4.0	0.0	24	
Males of the general population	24	2.5	2.9	3.0	0.0	10	0.3360**
Females of the general population	26	6.6	6.7	4.0	0.0	24	
Hypothyroidism	48	14.8	15.9	10.0	0.0	67.5	0.0003* 0.0273**
Hashimoto	35	31.1	16.3	27.2	13.6	88.3	0.0000* 0.0000**

*derived from Mann-Whitney test, when the group was compared with the general population group. **derived from Mann-Whitney test, when the group was compared with female individuals of the general population.

**Table 2:** Prevalence of anti-Neu5Gc positive samples.

	*n*	Positive %	Odd ratio	95% CI	P*
General population	50	6.0			
Hypothyroidism	48	42.1	0.089	0.024–0.381	<0.000
Hashimoto	35	97.1	0.002	0.0002–0.0188	<0.000

*Fisher's exact test.

**Table 3:** Concentration of anti-Neu5Gc IgM antibodies.

	*n*	Mean value (*μ*g/mL)	SD	Median value (*μ*g/mL)	Minimum (*μ*g/mL)	Maximum (*μ*g/mL)	P*
General population	50	2.5	2.7	1.6	1	7.9	
Hypothyroidism	48	3.3	2.8	1.3	2.2	12.0	0.00285
Hashimoto	35	5.0	3.1	0.1	4.2	12.6	0.00080

*derived from Mann-Whitney test (two tailed), when the group was compared with the general population group.

**Table 4:** Concentration of anti-Neu5Gc IgA antibodies.

	*n*	Mean value (*μ*g/mL)	SD	Median value (*μ*g/mL)	Minimum (*μ*g/mL)	Maximum (*μ*g/mL)	P*
General population	50	2.3	0.8	2.0	1.3	3.5	
Hypothyroidism	48	2.9	3.8	1.9	0.5	23.7	0.53024
Hashimoto	35	2.0	3.5	1.2	0.2	21.4	0.00285

*derived from Mann-Whitney test (two tailed), when the group was compared with general population group.

## Abbreviations

Neu5Gc: N-Glycolylneuraminic acid
Antithyroglobulin: Anti-TG
Antithyroid peroxidase: Anti-TPO
HRP: Horseradish peroxidase
Ab: Antibody
PBS: Phosphate buffer saline
PBST: Phosphate buffer saline, 0.05% Tween-80
CRP: C-reactive protein.
